# Natural Cyclic Peptides as an Attractive Modality for Therapeutics: A Mini Review

**DOI:** 10.3390/molecules23082080

**Published:** 2018-08-20

**Authors:** Muna Ali Abdalla, Lyndy J. McGaw

**Affiliations:** 1 Phytomedicine Programme, Department of Paraclinical Sciences, University of Pretoria, Private Bag X04, Onderstepoort 0110, South Africa; lyndy.mcgaw@up.ac.za; 2 Department of Food Science and Technology, Faculty of Agriculture, University of Khartoum, Khartoum North 13314, Sudan

**Keywords:** cyclic peptides, microorganisms, sponges, therapeutic agents, clinical trials, anticancer activity, antimicrobial activity, antiviral activity

## Abstract

Peptides are important biomolecules which facilitate the understanding of complex biological processes, which in turn could be serendipitous biological targets for future drugs. They are classified as a unique therapeutic niche and will play an important role as fascinating agents in the pharmaceutical landscape. Until now, more than 40 cyclic peptide drugs are currently in the market, and approximately one new cyclopeptide drug enters the market annually on average. Interestingly, the majority of clinically approved cyclic peptides are derived from natural sources, such as peptide antibiotics and human peptide hormones. In this report, the importance of cyclic peptides is discussed, and their role in drug discovery as interesting therapeutic biomolecules will be highlighted. Recently isolated naturally occurring cyclic peptides from microorganisms, sponges, and other sources with a wide range of pharmacological properties are reviewed herein.

## 1. Introduction

Peptides are among the most important biomolecules in nature. This class of compounds has gained special attention due to its remarkable variety of structures and valuable functions. Peptides display enormous variation in terms of both structure and function as they act as neurotransmitters [1] or as signalling molecules in the immune response [2] and hormones [3]. For instance, in higher organisms, peptides can be expressed locally in a flexible manner [4] and under physiological conditions, and their short half-lives facilitate quick removal after they have fulfilled their functions [5]. Cyclic peptides are polypeptide chains which are formed by amide bonds in a circular sequence between proteinogenic or nonproteinogenic amino acids. Many cyclic peptides are found in nature and several of them have been synthesized in the laboratory.

Cyclic peptides can play key roles in various processes and have excellent potential as therapeutics. Examples of widely discovered cyclic peptide therapeutic agents are: the antibiotics vancomycin, daptomycin, and polymyxin B; the hormone analogues oxytocin, octreotide, and vasopressin; and the immunosuppressant cyclosporine [6,7].

Due to the favourable characteristics of cyclic peptides, such as low toxicity, good binding affinity, and target selectivity, they are attractive candidates for the development of therapeutics [8,9]. Cyclic peptides are more cell permeable and have better biological activity compared with their linear counterparts due to their reduced conformational flexibility [10,11]. The rigidity of cyclic peptides play an important role in decreasing the entropy term of the Gibbs free energy, allowing these molecules to bind to multiple unrelated classes of receptors with very high affinity [11]. The privileged structures of cyclic peptides facilitate resistance to hydrolysis by exopeptidases because of the lack of both amino and carboxyl termini. Additionally, they are resistant to endopeptidases, as the structure is less flexible than their linear counterparts [12]. It is very interesting to note that cyclopeptides can adopt β-sheet-like arrangements, which can stack to create hollow tubular ensembles through the intermolecular hydrogen-bond network. Cyclic peptide nanoparticles and nanotubes have great potential for a wide range of biomedical applications [13,14,15]. Naturally occurring cyclic peptides have been isolated from plants [16], fungi [17,18], bacteria (including actinomycetes) [19,20,21], sponges [22], algae [23,24], and mammals [25].

Most of the clinically developed cyclopeptides are derived from natural products. Based on rational design, several recently approved powerful techniques were applied in addition to in vitro evolution, which enhanced the development of cyclic peptides synthesized de novo to targets for which nature does not offer solutions [8]. Some cyclic peptides from marine sources [26] have been approved by the Food and Drug Administration (FDA) such as ziconotide, a cyclic peptide isolated from the toxin of the cone snail species *Conus magus* [27]. Ziconotide is an analgesic drug used for severe and chronic pain that works by selective blocking of N-type calcium channels which control neurotransmission at many synapses [28].

In this mini review, the medicinal significance of naturally occurring cyclic peptides and their important role as therapeutic agents and biochemical tools is documented. Recently isolated natural cyclic peptides with antibacterial, antifungal, anticancer, antiviral, and other biological activities documented within the last four years are discussed.

## 2. Examples of Previously Isolated Cyclic Peptides Used as Therapeutic Agents

Vancomycin, a glycopeptide antibiotic, is an inhibitor of cell wall synthesis in susceptible organisms [29,30]. In addition to daptomycin, vancomycin is a first-line antibiotic choice for methicillin-resistant *Staphylococcus aureus* (MRSA) bacteremia [31]. It is recommended for intravenous administration as a standard therapy for patients with *S. aureus* bacteremia in complicated skin, bloodstream infections, endocarditis, bone and joint infections, and meningitis [30,32]. Due to the growing incidence of vancomycin-resistant bacteria [33,34], recent research resulted in the discovery of a dipicolyl–vancomycin conjugate (Dipi-van). This conjugate can inhibit cell-wall biosynthesis and enhance in vitro activity by more than two orders of magnitude higher than that of vancomycin alone [35].

The hepta-*N*-methyl undecapeptide cyclosporin A was created by nature as an orally bioavailable peptide drug [36]. Cyclosporin A has high membrane permeability, allowing it to cross the cell membrane due to its several intramolecular hydrogen bonds which keep hydrophilic groups from the surface of the molecule [11]. Cyclosporin was approved in 1983 as an immunosuppressive therapeutic drug in patients undergoing organ and bone marrow transplants, subsequently revolutionizing organ transplantation [37].

Romidepsin (also called FK-228, FR-901228, and Istodax) is a potent and selective histone deacetylase (HDAC) inhibitor. It was discovered in the early 1990s while evaluating antimicrobial and antitumour activities of fermentation products [38]. It has impressive clinical responses in patients with relapsed/refractory peripheral T-cell lymphoma (PTCL), leading to its approval by the US FDA in 2011 as an effective drug for PTCL in patients who have received at least one prior therapy [39].

**Figure 1:**
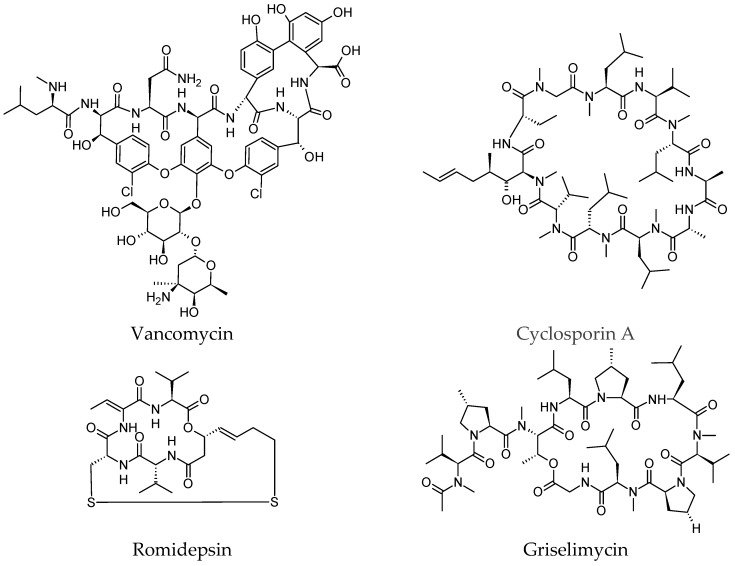
Previously discovered naturally occurring cyclic peptides.

Griselimycin is a macrocyclic poly-*N*-methylated depsipeptide discovered from *Streptomyces* bacterial cultures [40]. The new optimized derivatives of griselimycin exhibit striking activity in vitro and in vivo against *Mycobacterium tuberculosis* by inhibiting the DNA polymerase sliding clamp DnaN [41]. Griselimycin and its derivatives are potential targets to be taken through to the preclinical phase of drug development [42]. The structures of vancomycin, cyclosporin, romidepsin and griselimycin were drawn in Figure 1 and their medicinal significance were listed in Table 1.

## 3. Natural Cyclic Peptides and Analogues in Clinical Trials

More than 20 cyclic peptides have entered different phases in clinical trials. These compounds were developed for a wide range of medical conditions, including hematological diseases and cardiovascular disorders, many types of cancer, several infectious diseases, and endocrine/metabolic disorders. Many cyclic peptides, which are similar to those already approved, are analogues of natural products from microorganisms or human hormones. 

A number of natural cyclic peptides from marine sources are currently undergoing clinical evaluation, such as kahalalide F, an anticancer cyclic tridecapeptide from a sacoglossan mollusk, *Elysia rufescens*, and its diet alga *Bryopsis pennata*. Kahalalide F exhibited potent cytotoxic potential against a panel of human prostate and breast cancer cell lines, with IC_50_ ranging from 0.07 (PC3) to 0.28 µM [49,50]. Kahalalide F has reached clinical trials for the treatment of patients with solid tumors such as melanoma, non-small lung cancer, and hepatocellular carcinoma [51,52]. Due to a lack of antitumor activity, the trials were stopped. Fortunately, kahalalide F has appeared in recent investigations of advanced solid tumor therapy in a phase I clinical trial [53].

The anticancer compound elisidepsin is an analogue of kahalalide F, which exhibited in vitro activity against several tumor cell lines such as breast, colon, pancreas, prostate, and lung [54,55,56]. It showed promising results for further clinical studies of cancer therapy. Some clinical trials were submitted, including a study of elisidepsin in patients with advanced solid tumors [57]. The anticancer cyclic depsipeptide plitidepsin was isolated from the Mediterranean tunicate *Aplidium albicans* [58]. The chemical structure of plitidepsin closely resembles that of didemnin B, which has been submitted to clinical trials for many cancer treatments [59,60,61,62]. The clinical trials were ended due to severe fatigue and anaphylaxis experienced by patients [61,63]. Plitidepsin showed comparable levels to didemnin B in the in vitro anticancer activity to tumor cell lines [63,64]. It is currently undergoing phase I and II clinical trials. Results were announced in early 2016 following a small trial I for multiple myeloma [65]. A marine cyanobacterium, *Nostoc* sp. ATCC 53789 and GSV 224, afforded a depsipeptide cryptophycin, which is known as a potent fungicide [66]. It was discovered later that cryptophycin bound to the microtubule ends at the vinca-binding domain and inhibited the formation of the mitotic spindle. It showed strong anticancer activity against drug-resistant human cancer cell lines [67]. Cryptophycin-52, which is known as LY355703, is a synthetic derivative of the cryptophycin obtained by chemical synthesis. It was reported in a preclinical study [68] that cryptophycin-52 enhanced in vitro Bcl-2 hyperphosphorylation, cell cycle arrest, and reduced the growth of human non-small cell lung carcinoma cells. In Phase II clinical trials of cryptophycin-52, promising results of antitumor activity were found in patients with platinum-resistant advanced ovarian cancer [69] in addition to patients with advanced non-small cell lung cancer [70]. 

## 4. Recently Reported Naturally Occurring Bioactive Cyclopeptides Covering 2014–2018

### 4.1. Antibacterial Cyclic Peptides

The antibacterial cyclic peptides, pargamicins B, C, and D, were discovered from the fermentation broth of the soil actinomycete strain Amycolatopsis sp. ML1-hF [71].

Pargamicins A and C showed remarkable antibacterial activity against Gram-positive bacteria, including methicillin resistant *S. aureus* and vancomycin-resistant enterocci (VRE), which are the two most common healthcare-associated multidrug-resistant organisms. Moreover, the antibacterial activity of pargamicins B and D against these bacteria was weak. Pargamicins C and D, which have a polar group in the northern region of piperazic acid (Pip), showed 4- to 8-fold weaker activity against staphylococci than against enterococci. Additionally, pargamicins A and B exhibited the same activity against staphylococci and enterococci. The study reported that the presence of polar groups in the northern region of Pip may be responsible for the interaction with the staphylococcal membrane. Pargamicins were inactive against Gram-negative bacteria [55].

The antibacterial cyclic depsipeptide rakicidin F was isolated from the marine sponge-derived actinomycete strain *Streptomyces* sp. GKU 220 [72]. In the antimicrobial activity assay, rakicidin F had growth inhibitory activity against *Bacillus subtilis* and *Escherichia coli* at a dosage of 25 μg per disk.

**Figure 2:**
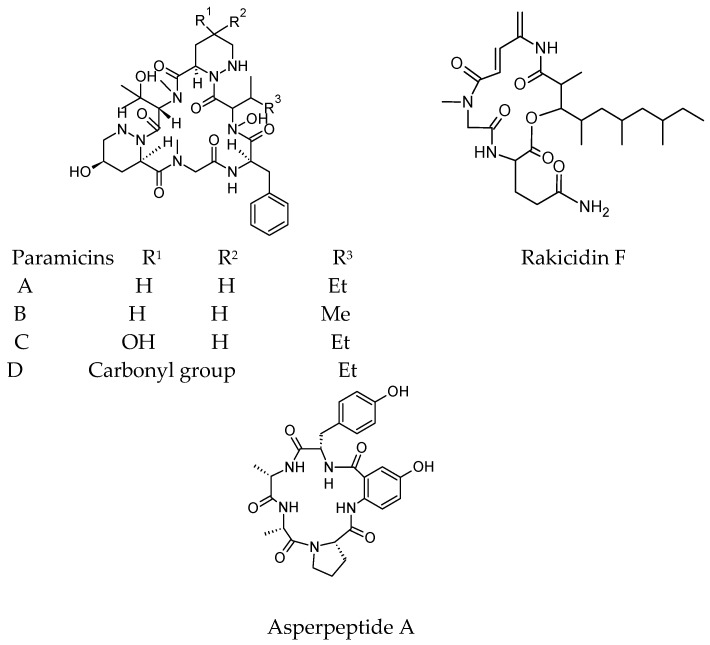
Recently isolated antibacterial cyclic peptides.

A cyclic pentapeptide named asperpeptide A cyclo(-Pro-Ala-Ala-Tyr-5-OHAA) was obtained from the gorgonian-derived fungus *Aspergillus* sp. XS-20090B15. Asperpeptide A showed antibacterial activity against *Bacillus cereus* and *Staphylococcus epidermidis* with the same MIC value of 12.5 μM [73]. Recently isolated antibacterial cyclic peptides were listed in Figure 2.

### 4.2. Antifungal Cyclic Peptides

An antifungal cyclic tetrapeptide, cyclo-(l-leucyl-trans-4-hydroxy-l-prolyl-d-leucyl-trans-4hydroxy-l-proline), was obtained from the coculture broth of two mangrove fungi, *Phomopsis* sp. K38 and *Alternaria* sp. E33. [74]. This compound showed moderate to high inhibitory activity against four crop-threatening fungi with MIC values of 220, 160, 130, and 250 µg/mL against *Gaeumannomyces graminis*, *Rhizoctonia cerealis*, *Helminthosporium sativum*, and *Fusarium graminearum*, respectively, compared with triadimefon. A cyclic depsilipopeptide colisporifungin was discovered from the liquid culture broths of the hitherto unstudied fungus *Colispora cavincola* following a *Candida albicans* whole-cell assay and a bioassay to discover the potential antifungal compound caspofungin [75].

A dose of 2 μg/mL of colisporifungin induced the antifungal activity of caspofungin against the pathogenic fungus *Aspergillus fumigatus*, which dropped the IC_50_ of caspofungin from ~33 to 6.2 nM, in a 5.3-fold increase in potency. Moreover, a dose of 1 μg/mL colisporifungin decreased the IC_50_ of caspofungin when tested against *C. albicans* [58].

Theonellamide G was isolated from the marine sponge *Theonella swinhoei* located in the Red Sea coast, Hurghada, Egypt. The bicyclic glycopeptide theonellamide G exhibited both antifungal and cytotoxic activities. It has potent antifungal potential against wild and amphotericin B-resistant strains of *C. albicans* with IC₅₀ of 4.49 and 2.0 μM, respectively. Moreover, it possesses cytotoxic activity against the human colon adenocarcinoma cell line (HCT-16) with IC₅₀ of 6.0 μM [76,77].

An antifungal cyclic hexapeptide named ASP2397 was produced by Malaysian leaf litter *Acremonium persicinum* MF-347833 [78]. This compound is similar to ferrichrome, a hydroxamate siderophore due to its ability to chelate aluminum ion. However, ASP2397 differs structurally from licensed antifungal agents such as amphotericin B, triazoles, and echinocandins. Four synthetic derivatives of ASP2397 were isolated from the culture broth and the metal-free form was converted chemically to other derivatives. Although ASP2397 differs structurally from the famous antifungal drugs such as amphotericin B, triazoles, and echinocandins. It exhibits potential activity against *Aspergillus* species and was found to be not cytotoxic to mammalian cells at concentrations as high as 50 μg·mL^−1^ and as also more soluble than the other derivative AS2529132. Therefore, ASP2397 was selected as a potential candidate for determination of its in vitro and in vivo activities.

**Figure 3:**
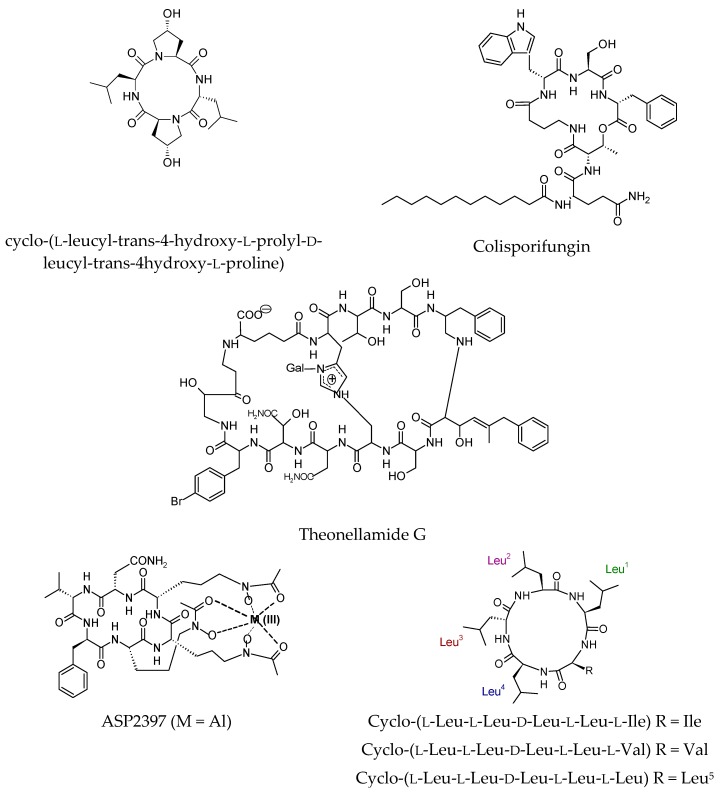
Recently isolated antifungal cyclic peptides.

Three cyclic pentapeptides named as Cyclo-(l-Leu-l-Leu-d-Leu-l-Leu-l-Ile), Cyclo-(l-Leu-l-Leu-d-Leu-l-Leu-l-Val), and Cyclo-(l-Leu-l-Leu-d-Leu-l-Leu-l-Leu) were delivered from the endophytic fungus *Fusarium decemcellulare* LG53, harbored in a Chinese medicinal plant *Mahonia fortunei* [79]. The three pentapeptides possessed moderate inhibitory effects towards three plant pathogenic fungi including *Aphanomyces cochlioides*, *Pythium ultimum*, and *Rhizoctonia solani*. Recently isolated antifungal cyclic peptides were drawn in Figure 3.

### 4.3. Anticancer Cyclic Peptides

Two cyclotetrapeptides, named cyclo-(Leu-Pro-Ile-Pro) and cyclo(Tyr-Pro-Phe-Gly), were isolated from the deep-sea bacterium *Bacillus amyloliquefaciens* GAS 00152 collected from the South China Sea deep-sea sediment [17,80].

The cytotoxicities of cyclo-(Leu-Pro-Ile-Pro) and cyclo(Tyr-Pro-Phe-Gly) were assayed in vitro against the HepG2 and HeLa cell lines using the MTT method. Cyclo-(Leu-Pro-Ile-Pro) was cytotoxic with IC_50_ values of 26.6 and 34.7 µM, respectively. The values of cyclo(Tyr-Pro-Phe-Gly) were 38.2 and 46.1 µM, respectively.

Wewakazole B, an antitumor cyanobactin, was isolated from the cyanobacterium *Moorea producens* collected in the Red Sea. The structure was elucidated by means of NMR and MS techniques [81]. Wewakazole B showed cytotoxic activity against human MCF-7 breast cancer cells (IC_50_ = 0.58 μM) and human H460 lung cancer cells (IC_50_ = 1.0 μM) and was inactive in a siderophore assay.

The cyclotripeptide psychrophilin E was produced by a coculture of two marine alga-derived fungal strains of *Aspergillus* [82]. It showed selective antiproliferative activity towards the HCT116 (colon) cell line with IC_50_ values of 28.5 µM compared with cisplatin as a positive control (IC_50_ 33.4 µM). A cyclohexapeptide, similanamide, was obtained from the culture of the marine sponge-associated fungus *Aspergillus similanensis* KUFA 0013 [83]. Similanamide showed weak anticancer activity against MCF-7 (breast adenocarcinoma, GI_50_ = 125 ± 0), NCI-H460 (non-small cell lung cancer, GI_50_ = 117.50 ± 3.55), and A373 (melanoma, GI_50_ = 115 ± 7.07) cell lines, and it was inactive in the antibacterial bioassay.

A cyclic pentapeptide known as disulfide cyclo-(Leu-Val-Ile-Cys-Cys) and named malformin E, along with 13 known cyclic dipeptides, was obtained from the culture broth of the endophytic fungus *Aspergillus tamarii* harbored by *Ficus carica*. Malformin had potent anticancer activities towards the human cancer cell strains MCF-7 and A549, with IC_50_ values of 0.65 and 2.42 μM, respectively. It also showed potential antibacterial activities against *B. subtilis*, *S. aureus*, *Pseudomonas aeruginosa*, and *E. coli* and antifungal potential against *Penicillium chrysogenum*, *C. albicans*, and *Fusarium solani*, with MIC values of 0.91, 0.45, 1.82, 0.91, 3.62, 7.24, and 7.24 μM, respectively [84]. Unprecedented highly *N*-methylated cyclic octadecapeptides named as gymnopeptides A and B were isolated from the mushroom *Gymnopus fusipes*. Gymnopeptides A and B showed impressive antiproliferative activity on several human cancer cell lines including cervical (HeLa), skin epidermoid (A431), and breast (T47D, MCF7, and MDAMB-231) cell lines, with nanomolar IC_50_ values [85]. Although both compounds were at least two orders of magnitude more efficient than that of the reference compound cisplatin, the study reported that gymnopeptide B is more potent than gymnopeptide A. These compounds highlighted the importance of mushroom cyclopeptides [86].

The cyclic tetrapeptides pseudoxylallemycins A–F, with rare allenyl modifications, were obtained from *Pseudoxylaria* sp. X802, which is known as a competitor of Fungus-Growing Termite Cultivars [87]. Pseudoxylallemycins A–F have antiproliferative activity towards human umbilical vein endothelial cells (HUVEC) and K-562 cell lines, with GI_50_ values of 4.2 μg/mL(K-562 forpseudoxylallemycin C) to 42.8 μg/mL (K-562 for pseudoxylallemycin D), in addition to cytotoxic activity (HeLa cells) with a CC_50_ value as low as 10.3 μg/mL (for pseudoxylallemycin C). Additionally, they showed antimicrobial activity against the Gram-negative human-pathogenic *P. aeruginosa*.

The marine sponge *Reniochalina stalagmitis*, collected from Yongxing Island in the South China Sea, delivered five cyclic peptides (including four heptapeptides and one octapeptide), named reniochalistatins A–E [88]. Reniochalistatins A–E were tested towards five different human cancer cell lines (RPMI-8226, MGC-803, HL-60, HepG2, and HeLa). Interestingly, the cyclic octapeptide reniochalistatin E exhibited biological activity towards myeloma RPMI-8226 and gastric MGC-803 cells with IC_50_ values of 4.9 and 9.7 μM, respectively. Additionally, it was inactive against leukemia HL-60 and hepatoma HepG2 (IC_50_ > 20.0 μM) and cervical HeLa (IC_50_ > 17.3 μM) cells.

**Figure 4:**
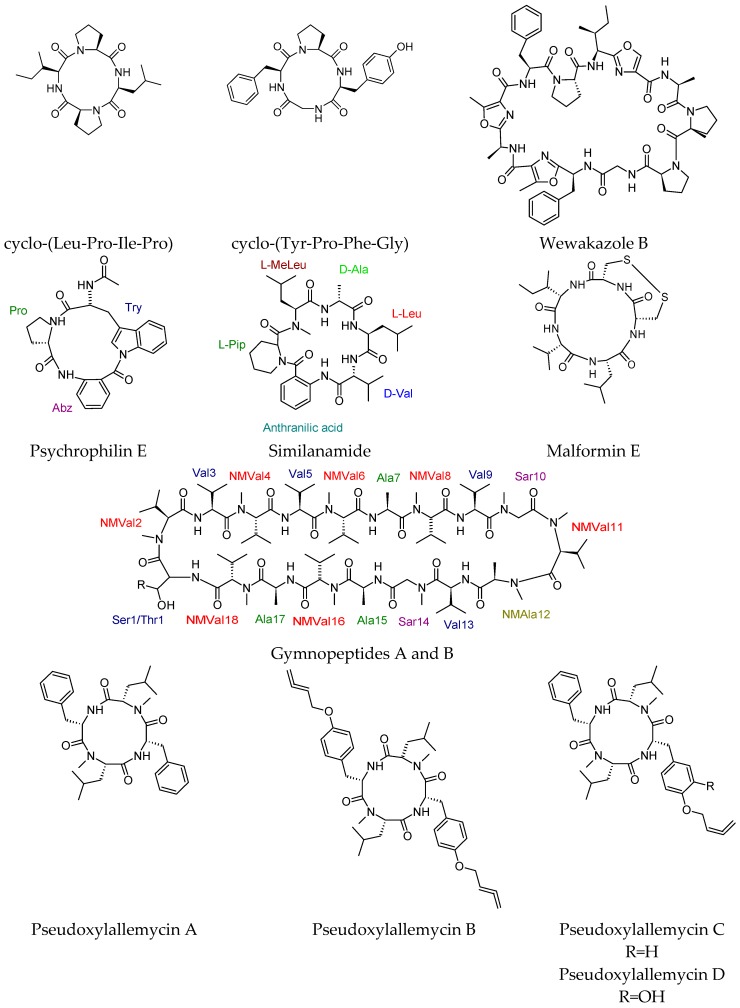

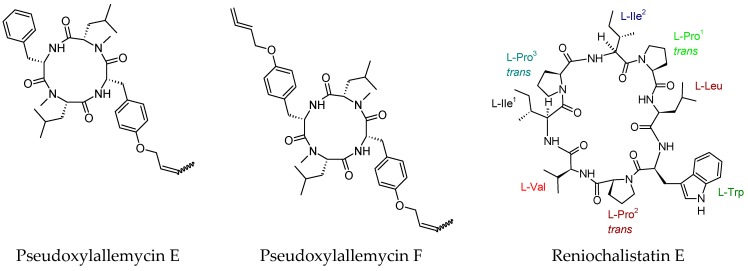
Recently isolated anticancer cyclic peptides.

The four heptapeptides (A–D) were inactive against the tested cell lines. This report has drawn attention to the marine secondary metabolites from the sponges of the genus *Reniochalina*, which was previously dominated by dihydrothiopyranone, fatty acids, and acetylenic alcohols [89,90,91]. Recently isolated anticancer cyclic peptides were listed in Figure 4.

### 4.4. Recently Antiviral Isolated Cyclic Peptides

**Figure 5:**
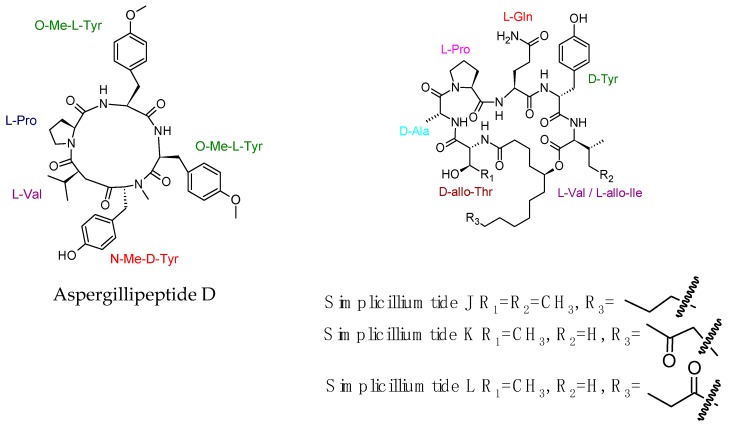

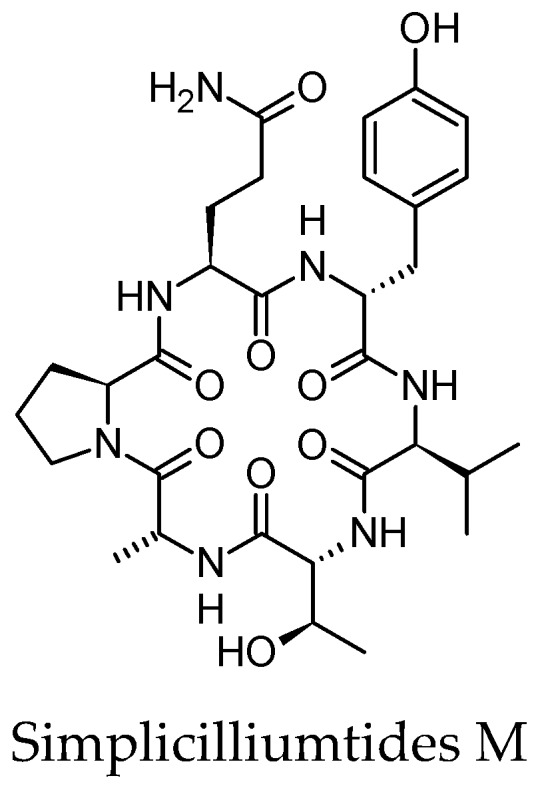
Recently isolated antiviral cyclic peptides.

A pentacyclic peptide named aspergillipeptide D was isolated from a culture broth of the marine gorgonian-derived fungus *Aspergillus* sp. SCSIO 41501 [92]. Aspergillipeptide D exhibited good antiviral activity towards herpes simplex virus type 1 (HSV-1), with IC_50_ values of 9.5 µM under their noncytotoxic concentrations (TC_0_) against a Vero cell line with TC_0_ and TC_50_ values of 81.9 and 204.4 µM, respectively. It also showed antiviral activity towards acyclovir-resistant clinical isolates of HSV-1-106 and HSV-1-153 at a concentration of 12.5 µM with about 50% inhibition rate. The cyclic peptides simplicilliumtides J–M were identified along with a linear peptide and other known analogues verlamelins A and B from the deep-sea-derived fungal strain *Simplicillium obclavatum* EIODSF 020. Simplicilliumtide K exhibited significant antiviral activity toward HSV-1 with IC_50_ value of 14.0 μM, and it also showed antifungal activity against *Aspergillus versicolor* and *Curvularia australiensis* [93]. Recently isolated antiviral cyclic peptides were drawn in Figure 5.

### 4.5. Cyclic Peptides with Diverse Biological Activities

Bioactive cyclic hexapeptides anabaenopeptins (**1**–**5**) were isolated from an extract of Baltic Sea cyanobacterial bloom material contained of *Nodularia spumigena* (50%), *Aphanizomenon flos-aquae* (40%), and *Dolichospermum* spp. (10%) by preparative reversed-phase high performance liquid chromatography (HPLC) [94]. The five anabaenopeptins showed inhibitory activity against carboxypeptidase A (apart from one anabaenopeptin variant) in addition to protein phosphatase 1 with different potency. None of the compounds exhibit inhibitory activity against chymotrypsin, trypsin, and thrombin.

Two cyclic peptides, named pentaminomycins A and B, were obtained from cultures of *Streptomyces* sp. RK88-1441. Pentaminomycin A inhibited α-MSH-stimulated melanin synthesis by slowing the expression of melanogenic enzymes such as tyrosinase, tyrosinase-related protein-1 (TRP-1), and tyrosinase-related protein-2 (TRP-2) [95]. 

The marine-derived fungus *Stachylidium* sp. was isolated from the sponge *Callyspongia* sp. cf. *C. flammea* afforded two cyclic tetrapeptides, bearing a very rare amino acid 3-(3-furyl)-alanine, and these were named endolides A and B. Radioligand binding assays were performed and endolide A had affinity to the vasopressin receptor 1A with a Ki of 7.04; additionally, endolide B showed affinity toward the serotonin receptor 5HT2b with a Ki of 0.77 μM [96].

**Figure 6:**
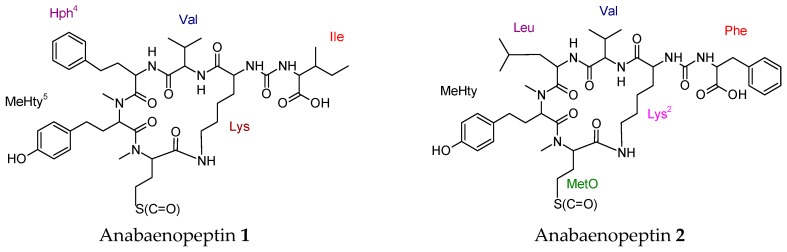

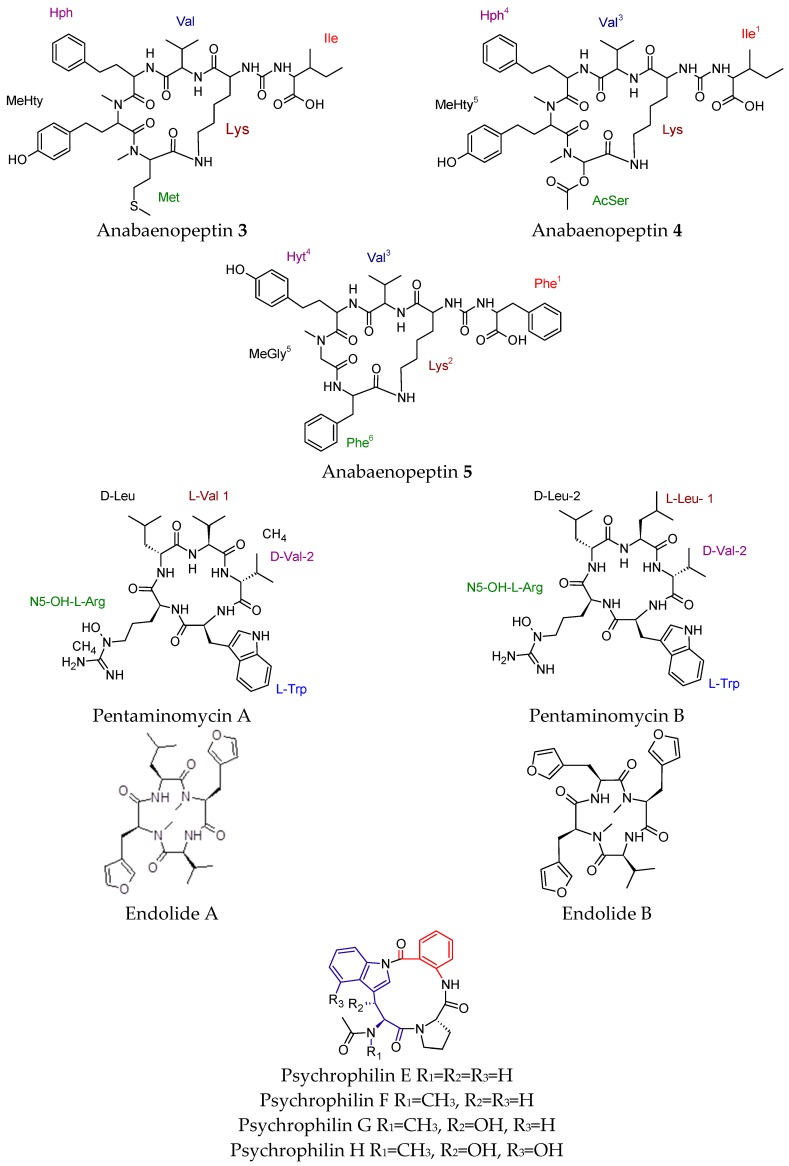
Recently isolated cyclic peptides with diverse biological activities.

Four cyclic tetrapeptides, named as psychrophilins E-H, were isolated from the marine-derived fungus *Aspergillus versicolor* ZLN-60. They are rare fungal cyclic peptides, which have amide groups consisting of anthranilic acid and indole moieties in the macrocycle. Psychrophilin G exhibited potent lipid-lowering effects. Recently isolated cyclic peptides with diverse biological activities were drawn in Figure 6.

### 4.6. Cyclic Peptides with No Reported Biological Activity

Cyclic tetrapeptides auxarthrides A and B were obtained from cultures of the coprophilous fungus *Auxarthron pseudauxarthron* [97]. These compounds were inactive in the antifungal bioassay and against cancer cell lines. A cyclic heptapeptide, talarolide A, was isolated from an Australian marine tunicate-associated fungus, *Talaromyces* sp. (CMB TU011), by following a miniaturized 24-well plate microbioreactor approach. The compound was tested for anticancer activity towards human embryonic kidney (HEK-293) and colorectal (SW-620) adenocarcinoma cells. It was also tested for antimicrobial potential against *Candida albicans* (ATCC 90028), the Gram-negative bacteria *Escherichia coli* (ATCC 11775) and *Pseudomonas aeruginosa* (ATCC 10145), and the Gram-positive bacteria *Staphylococcus aureus* (ATCC 9144 and ATCC 25923) and *Bacillus subtilis* (ATCC 6633 and ATCC 6051), but unfortunately, it was inactive (IC_50_ > 30 μM). The study suggested that it may have a specialized ecological purpose [98]. The endophytic fungus *Penicillium tropicum*, which harboured *Sapium ellipticum,* afforded a cyclohexapeptide penitropeptide. The compound was tested for its cytotoxic and antibacterial activities, but it was inactive [99].

The cyclotetrapeptides sartoryglabramides A and B were isolated from the marine sponge-associated fungus *Neosartorya glabra* KUFA 0702. Both compounds were inactive in the antimicrobial bioassay [82]. Cyclic heptapeptides, mortiamides A–D, were isolated from a *Mortierella* sp. isolate which was found in marine sediment collected from the intertidal zone of Frobisher Bay, Nunavut, Canada. None of these compounds exhibited antimicrobial or cytoxic activities [100].

**Figure 7:**
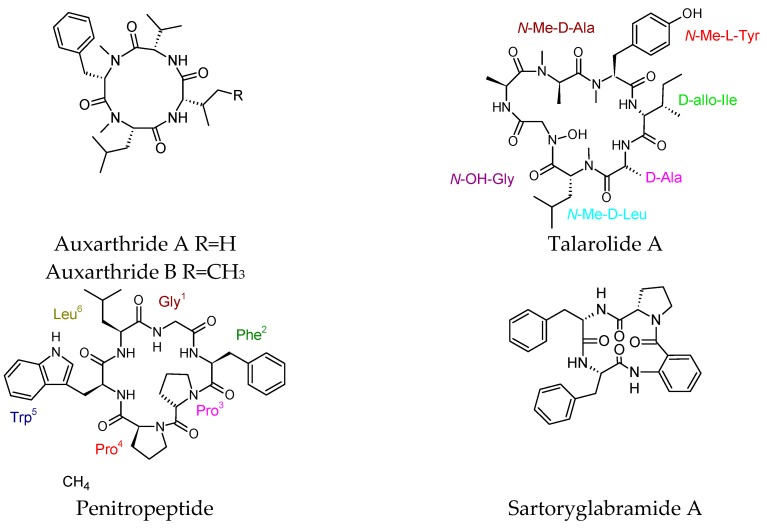

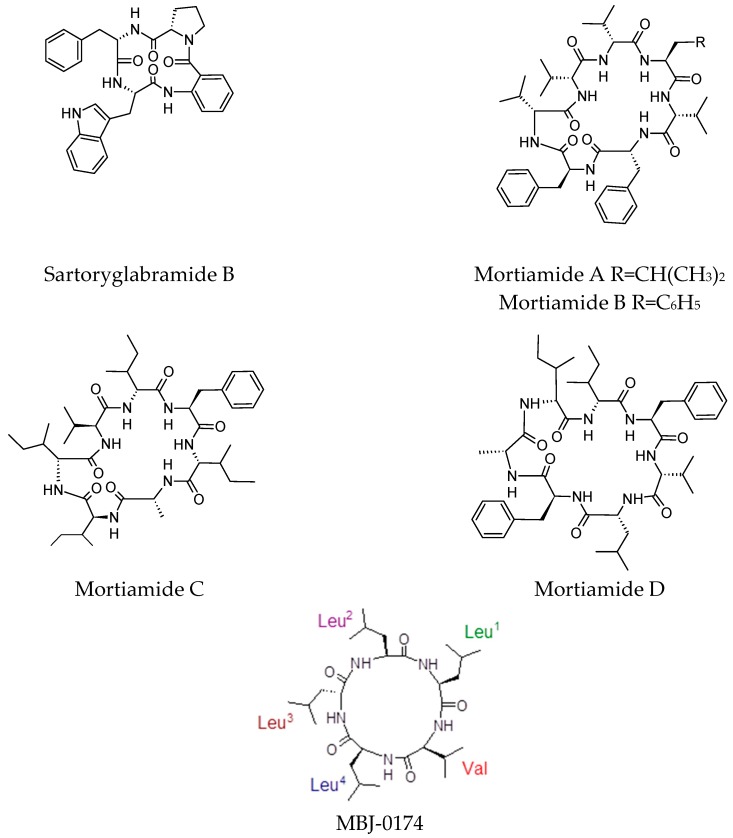
Recently isolated cyclic peptides with no reported biological activity.

A cyclic pentapeptide named MBJ-0174 was isolated, together with a linear peptide MBJ-0173, from the culture broth of *Mortierella alpina* f28740 derived from a soil sample collected in Ise, Japan [101]. The compound did not show antimicrobial or cytotoxic activities. Recently isolated cyclic peptides with no reported biological activity were drawn in Figure 7.

## 5. Conclusions and Future Perspectives

For a long time, much attention has been paid to cyclic peptides derived from natural sources due to their therapeutic potential. In this context, microbial cyclic peptides have brought chemical templates for clinically potent lead compounds to the attention of the pharmaceutical industry. Several cyclic peptides and analogues derived from marine sources exhibited different biological activities, including anticancer, antimicrobial, antiparasitic, anti-inflammation, antiproliferative, and anti-hypertensive properties. Cyanobacteria too are excellent sources of structurally diverse marine cyclic peptides with a broad range of pharmacological properties. It is important to mention that marine peptides and analogues have high commercial value and have entered the nutraceutical and pharmaceutical markets. Several of them are in different stages of the clinical and preclinical pipeline. Various recently isolated cyclic peptides obtained from fungi and marine sources exhibited interesting biological activities. Examples of these include potent anticancer compounds active against several cancer cell lines and gymnopeptides A and B from the mushroom *G. fusipes*. The antibacterial pseudoxylallemycins A–F from *Pseudoxylaria* sp. X802 with rare allenyl modifications have interesting properties. The antifungal cyclic hexapeptide ASP2397 from *A. persicinum* was reported to be a potential therapeutic drug, but further studies are required to elucidate its antifungal mechanisms as well as its derivatives. An interesting cyclic pentapeptide, a disulfide cyclo-(Leu-Val-Ile-Cys-Cys) malformin E, isolated from the culture broth of endophytic fungus *A. tamarii* exhibited potent anticancer as well as antimicrobial activities. The marine-derived fungus *Stachylidium* sp. delivered endolides A and B, which showed affinity to the vasopressin receptor 1A and serotonin receptor 5HT2b, respectively, in radioligand binding assays. Interestingly, psychrophilins E-H, which were discovered from the marine-derived fungus *Aspergillus versicolor* ZLN-60, are rare fungal cyclic peptides, which have amide groups consisting of anthranilic acid and indole moities in the macrocycle. Psychrophilin G exhibited potent lipid-lowering effects.

Hence, fractionation and structure elucidation of active secondary metabolites is urgently needed. It is important to note that isolation of bioactive natural products has become more difficult because traditional methods typically lead to known metabolites. Developing new dereplication procedures should be followed in addition to employing novel techniques and strategies in chemical, biological, and biotechnological screening. It is very important to describe a systematic assessment of natural species abundance as a constructive step in the discovery of novel cyclic peptides. Successful approaches and strategies such as discovery of new taxa, unculturable microorganisms, mixed fermentation, and genome mining could have an important impact on the discovery of interesting and useful cyclic peptides.

## Figures and Tables

**Table 1:** Previously discovered naturally occurring cyclopeptides and their medicinal significance.

Cyclic Peptide	Natural Source	Medicinal Significance	Mechanism of Action
Vancomycin	*Amycolatopsis orientalis*	An antibiotic mostly active against Gram-positive microorganisms, including MRSA but not vancomycin-resistant *Enterococcus* (VRE). It is used for bacterial prophylaxis in neurological, orthopedic, and vascular surgery. For patients who are allergic to penicillins and cephalosporins, it can be used as an alternative antibiotic [43].	Inhibiting cell wall synthesis of bacteria by binding to the building blocks of peptidoglycan monomers of *N*-acetylmuramic acid and *N*-acetylglucosamine and blocking cross-linking of the peptidoglycan layer [44].
Cyclosporin A	The fungus *Tolypocladium inflatum*	Has potent immunosuppressive properties. It prevents graft-versus-host disease following transplantation, rejection of kidney, heart, and liver transplants [45].	It blocks the transcription of cytokine genes in activated T cells via the calcineurin-phosphatase pathway [46]. It binds to the cytosolic protein cyclophilin of T cells and consequently leads to reduced T-cell function. It also binds to the cyclophilin D protein that constitutes part of the mitochondrial permeability transition pore (MPTP), which causes movement of calcium ions (Ca^2+^) into the mitochondria and causes the contraction of the muscle cells (heart) [47].
Romidepsin	*Chromobacterium violaceum*	A potent antitumor drug that reverses the malignancy of tumorigenic cell lines and induces apoptosis in malignant cell lines.	Promotes acetylated histones H3 and H4 in the peroxiredoxin 1 (Prdx1) promoter (as a tumor suppressor), thus activating Prdx1 expression in tumor tissues and inhibiting tumour growth [48].
Griselimycin	*Streptomyces* bacteria	Griselimycin derivatives showed antibiotic activity in addition to oral bioavailability, absorption, and antitubercular activity [40]. Griselimycin exhibited formidable pharmacokinetic properties for its chemical class and size.	Prevents DNA replication (known as sliding clamp) by inhibiting the interaction of the replicative DNA polymerase with the DNA polymerase beta subunit [41].
